# Self-Reported Nonadherence to Medication Is Not Associated with Health-Related Quality of Life in Parkinson’s Disease

**DOI:** 10.3390/brainsci11020273

**Published:** 2021-02-22

**Authors:** Hannah M. Zipprich, Sarah Mendorf, Thomas Lehmann, Tino Prell

**Affiliations:** 1 Department of Neurology, Jena University Hospital, 07747 Jena, Germany; sarah.mendorf@med.uni-jena.de; 2 Center for Clinical Studies, Jena University Hospital, 07747 Jena, Germany; thomas.lehmann@med.uni-jena.de; 3 Department of Neurology and Center for Healthy Ageing, Jena University Hospital, 07747 Jena, Germany; tino.prell@med.uni-jena.de

**Keywords:** nonadherence, Parkinson’s disease, unified Parkinson’s disease rating scale, German Stendal Adherence with Medication Score, quality of life, motor impairment

## Abstract

Nonadherence is a growing issue in the treatment of Parkinson’s disease (PD). Many factors are known to influence nonadherence, but little is known about the influence of quality of life (QoL). Detailed clinical data were obtained from 164 patients with PD using the Parkinson’s Disease Questionnaire-39 (PDQ-39) and the German Stendal Adherence with Medication Score (SAMS). Descriptive statistics were used to identify reasons for nonadherence, and multivariable linear models were used to study associations between QoL and clinical parameters as well as nonadherence. Multivariate analysis of variance (MANOVA) and multivariate analysis of covariance (MANCOVA) were used to study the effect of the SAMS on PDQ domains and other medical covariates. The results showed that 10.4% (*n* = 17) of patients were fully adherent, 66.4% (*n* = 109) were moderately nonadherent, and 23.2% (*n* = 38) were nonadherent. Nonadherence was associated with male gender, lower Montreal Cognitive Assessment (MoCA) score, higher non-motor symptoms questionnaire (NMS-Quest) score, greater number of medications per day (an indicator of comorbidity), and higher Beck Depression Inventory (BDI) score. QoL was correlated with male gender, lower MoCA score, higher NMS-Quest score, more comorbidities, and higher BDI score, but was not correlated with nonadherence.

## 1. Introduction

Nonadherence to medication is a common and serious issue in the care of people with Parkinson’s disease (PD). The factors associated with nonadherence to medication include patient characteristics, disease-related factors, financial and health system barriers, patient–provider relationships, and treatment-related factors. Patient-related factors can be intentional (when the patient purposefully decides not to take the prescribed medication) or unintentional (when the patient cannot follow the recommendations). In several cohorts with specific diseases, nonadherence was found to be associated with health-related quality of life (HRQoL) [1,2,3]. This is why QoL can improve following interventions to improve medication adherence [1]. So far, only a few cross-sectional studies have examined the association between adherence and HRQoL in PD patients. Although all these studies used the PD Questionnaire (PDQ-8 or PDQ-39) to assess HRQoL, they used different tools, such as electronic monitoring bottles, to measure nonadherence. Grosset et al. observed that in 54 PD subjects, all eight domains of PDQ-39 correlated with medicine usage, with the association being strongest for social support [4]. In another study of 112 PD subjects, the PDQ-39 summary index did not significantly differ between patients with suboptimal and those with satisfactory adherence. However, total adherence to therapy had a significant negative association with both the Unified Parkinson’s Disease Rating Scale (UPDRS) III score and the PDQ-39 mobility domain [5]. In another study of 124 PD subjects, the 8-Item Morisky Medication Adherence Scale (a common self-report adherence questionnaire) correlated weakly with the PDQ-8 summary index; however, this significant correlation did not survive correction for other clinical variables in stepwise multiple regression [6]. A major limitation of these three studies is that patients with cognitive impairment were not considered. This limits the generalizability of these data in a disease in which the cumulative incidence of dementia reaches 83% 20 years after diagnosis [7].

In the current study, we aimed to provide additional data to determine whether self-reported nonadherence is related to HRQoL in PD. For this purpose, we analyzed the relationship between a detailed German self-report adherence questionnaire and the PDQ-39 in a sample of 164 PD patients. Because a holistic view is especially needed for elderly people, the assessed adherence referred to all medication and was not restricted to PD medication.

## 2. Methods

### 2.1. Subjects

This observational study was approved by the local ethics committee of the Jena University Hospital, Germany. All subjects gave written informed consent in accordance with the Declaration of Helsinki. Patients with PD according to the Movement Disorder Society (MDS) diagnostic criteria [8,9] were consecutively recruited from January 2019 until the beginning of the COVID-19 pandemic in March 2020 from the movement disorder ward and the outpatient clinic of the Department of Neurology of the Jena University Hospital. Patients with delirium and severe dementia with an inability to understand and complete a questionnaire were excluded. All tests were conducted during the medication ON phase. The data were collected by trained research staff (students of psychology) and a PD nurse specialist. After a short introduction to the aims and methods of the study, the Montreal Cognitive Assessment (MoCA) [10] was performed. This allowed us to form a realistic impression of whether the patient was able to understand and complete a valid questionnaire. None of the patients had PD dementia. However, it is important to note that several patients had a MoCA < 21 points because of visual impairments due to eye problems, problems in fine motor skills, or the inability to hold a pen.

### 2.2. Assessment

The following demographic and clinical data were collected: age, gender, marital status, level of education, total daily number of medications administered in any pharmaceutical form (as an indicator of comorbidity), Beck Depression Inventory (BDI) II score, the MDS-sponsored revision of the UPDRS III (MDS-UPDRS III), the revised non-motor symptoms questionnaire (NMS-Quest), and, for HRQoL, the PDQ-39. The PDQ-39 is composed of 39 items grouped in eight domains. Each item is scored from 0 (never) to 4 (always). Domains for mobility, activities of daily living, emotional well-being, stigma, social support, cognition, communication, and bodily discomfort and a summary index representing the global HRQoL were calculated, with higher scores representing worse HRQoL. Adherence was assessed by the self-reported German Stendal Adherence to Medication Score (SAMS). The SAMS includes 18 questions forming a cumulative scale (0–72), in which 0 indicates complete adherence and 72 indicates complete nonadherence [11]. The full SAMS and the handbook are available online (CC BY NC 3.0 license) [12].

### 2.3. Statistical Analyses

The SPSS statistical computer package (version 25.0; IBM Corporation, USA) was used for all statistical analyses. Values are given as mean and standard deviation or median and interquartile range. Categorical variables are presented as numbers or percentages. For all analyses, *p* < 0.05 was considered to indicate statistical significance.

It is generally considered that suboptimal adherence becomes clinically meaningful when <80% of the prescribed medication is taken [13,14]. This leads to a study- and sample-specific SAMS cutoff of 11 points for clinically meaningful nonadherence. The patients were then categorized as fully adherent (SAMS = 0), moderately nonadherent (SAMS 1–10.9), or nonadherent (SAMS ≥ 11).

In the first step, we described the cohort using descriptive statistics. Normal distribution was determined by the Shapiro–Wilk test. Correlation analyses were performed using Spearman’s correlation for skewed data and the Kruskal–Wallis test for group comparisons. To explore predictors of nonadherence, a multivariable linear regression was performed with the SAMS as the dependent variable and several clinical variables (age, educational level, BDI, MoCA, MDS-UPDRS III, NMS-Quest, disease duration, number of medications per day, assessment in outpatient or inpatient setting) as independent variables (stepwise forward, Akaike information criterion). To study the association between HRQoL and adherence, multivariable linear regressions were performed with the PDQ-39 summary index or PDQ-39 domains as the dependent variable and the SAMS and the clinical cofactors (as above) as independent variables (stepwise forward, Akaike information criterion). Multivariate analysis of variance (MANOVA) was used to study the relationship between the SAMS and the PDQ-39 domains. Multivariate analysis of covariance (MANCOVA) was used to adjust these findings for other clinical factors. There was homogeneity of the error variances for all PDQ-39 domains, as assessed by Levene’s test, and homogeneity of the covariances, as assessed by Box’s test (*p* > 0.001).

## 3. Results

The final sample included 164 patients with PD, of whom 64 (39%) were female, with a mean age of 71 ± 9 years. The majority of the patients were married and had completed middle or high school. Detailed clinical data are provided in Table 1. The mean total SAMS was 6.7 ± 5.8 points. According to the SAMS, 17 (10.4%) of the patients were fully adherent (SAMS = 0), 109 (66.4%) were moderately nonadherent (SAMS 1–10.9), and 38 (23.2%) showed clinically meaningful nonadherence (SAMS ≥ 11). Nonadherence was associated with male gender, lower MoCA, higher NMS-Quest, higher number of medications per day, and higher BDI (Table S1).

**Figure 1:**
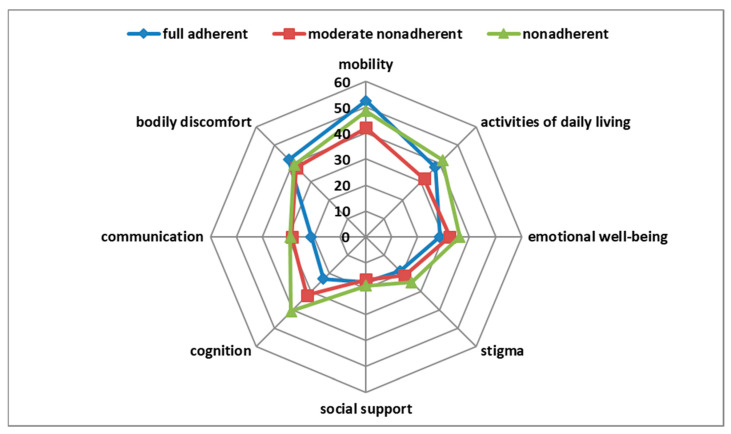
Correlation of adherence with Parkinson’s Disease Questionnaire-39 (PDQ-39) domains.

In univariate analysis, the SAMS correlated weakly with the PDQ-39 domain cognition (*r* = 0.21, *p* = 0.007), but not with the other domains and not with the PDQ-39 summary index. Fully adherent, moderately nonadherent, and nonadherent patients differed only in the PDQ-39 domain cognition (*p* = 0.005) (Figure 1). After correction for other clinical variables in multivariable regression, the SAMS was not a significant predictor of the PDQ-39 domain cognition (Table S1). As outlined above, the data collection was terminated by the COVID-19 pandemic. Fortunately, a post-hoc power analysis revealed: with a coefficient of determination of R^2^ = 0.48 (for PDQ-39 cognition domain), a statistical power of 0.95, and a significance level of α = 0.01, a sample size of *n* = 34 would be needed for a significant overall model with four predictors (BDI, SAMS, MoCA, NMS-Q). Therefore, we believe that our sample size is sufficient.

One-way MANOVA showed a statistically significant difference between patients with different degrees of adherence (fully adherent, moderately nonadherent, nonadherent) on the combined PDQ-39 domains (F(16,308) = 1.85, *p* = 0.025, partial η^2^ = 0.088, Wilk’s λ = 0.83). Post hoc univariate ANOVAs were conducted for every dependent variable. The results showed a statistically significant difference between patients with different degrees of adherence for the PDQ-39 domain cognition (F(2161) = 5.42, *p* = 0.005, partial η^2^ = 0.063), but not for the other PDQ-39 domains. Tukey Honestly Significant Difference post hoc analysis on the PDQ-39 domain cognition revealed a significant difference between the fully adherent and the nonadherent group (*p* = 0.006; MDiff = −17.62; 95% CI−30.9, −4.31) and between the moderately nonadherent and the nonadherent group (*p* = 0.047; MDiff = −8.68; 95% CI−17.3, −0.09), but not between the fully adherent and the moderately nonadherent group (*p* = 0.18). MANCOVA was conducted to examine whether other medical covariates could account for these findings. Therefore, the variables known to be related to HRQoL (BDI, MoCA, and NMS-Quest) (Table S1) were included in the model as covariates. In this analysis, BDI (Wilk’s λ = 0.73, *p* < 0.001, partial η^2^ = 0.27) and NMS-Quest (Wilk’s λ = 0.55, *p* < 0.001, partial η^2^ = 0.45) were significant, but MoCA (*p* = 0.09) and the degree of adherence (adherent, moderately nonadherent, and nonadherent) (*p* = 0.13) were not significant.

## 4. Discussion

Our study showed that self-reported nonadherence was not associated with the PDQ-39 summary index, nor with the different domains of the PDQ-39. The weak correlation between the SAMS and the domain cognition did not survive correction for other clinical parameters. This extends the results of Straka et al., where the PDQ-8 summary index did not correlate with the 8-Item Morisky Medication Adherence Scale after correction for other clinical factors [5,6]. It can therefore be concluded that for patients with PD, other aspects are more relevant to HRQoL than adherence. We can only speculate if this is a special phenomenon in PD, which is defined by distinct motor and non-motor symptoms that strongly influence QoL. Nevertheless, it is interesting to note that in other disorders (e.g., hypertension, epilepsy, and heart failure), adherence to pharmacological treatment is commonly associated with better HRQoL [2,3,15,16,17,18]. The observed predictors of the PDQ-39 in our study, namely motor function, age, depression, and gender, are in line with earlier reports of people with PD [19,20,21,22].

The two studies by Grosset et al. mentioned above are comparable to our study only to a limited extent because electronic monitoring was used to measure adherence [3,4]. In addition, the patients in the studies by Grosset et al. were younger, less cognitively impaired, and subject to a different selection and enrolment procedure (Table S2). Therefore, the investigated cohorts are not well comparable.

Given the high prevalence of cognitive impairment in PD, this aspect should be considered in more detail. In our study, patients with manifest PD dementia or psychotic symptoms were excluded. The low MoCA in our patients is due to the fact that some of the patients could not complete formal items of the MoCA, for example, because of visual or motor problems. In our previous study, we observed no association between self-reported adherence and cognition in people with PD [23].

Our study has several limitations. When interpreting the results, one has to consider the limitations of the HRQoL measures that were used. HRQoL measures such as PDQ-39 are weighted toward health-related functions and therefore only cover one aspect of QoL. It is important to be aware that adherence and HRQoL are dynamic constructs that may change during the course of the disease. While one can hypothesize that nonadherence can cause poor HRQoL (i.e., worsening of PD symptoms due to missed doses), it is also possible that improved HRQoL may be a trigger for nonadherence (i.e., patients do not see the need for sustained treatment when symptoms have improved). Therefore, repeated measures of adherence and HRQoL are necessary to fully evaluate whether there is a potential dynamic relationship between adherence and HRQoL in PD. It is also noteworthy that the prevalence of nonadherence cannot be compared between subjective (e.g., SAMS) and objective (e.g., electronic pill monitoring) measurements because self-reports can overestimate adherence [24]. Additionally, we did not assess the types and dosages of medication taken, neither of medication related to PD nor to other conditions. To better understand the impact on PDQ-39 and HrQoL, it would be important to not only know the types and dosages of prescribed medication, but which medication was not taken by nonadherent patients as well. This should definitely be taken into account in future studies. 

## 5. Conclusions

Using an external clinical outcome (e.g., blood pressure, cardiovascular events) to validate an adherence scale seems to be a reasonable approach for carefully selected patients and distinct disorders. However, as demonstrated by our study, the PDQ-39 is probably not a suitable external clinical outcome to validate a self-report adherence scale.

## Figures and Tables

**Table 1:** Characteristics of the cohort.

Characteristic		*n*	%
Sex	Female	64	39.0
	Male	100	61.0
Marital status	Married	121	75.6
	Widowed or divorced	35	21.9
	Single	4	2.5
Educational level	Low	36	22.2
	Middle	57	35.2
	High	69	42.6
		**Mean**	**SD**
Number of medications		7.27	4.29
Age (years)		71.04	8.97
Disease duration (years)		9.41	5.90
Hoehn and Yahr stage (median, interquartile range)		3.0	0.0
BDI		11.39	6.64
MoCA		22.46	4.21
SAMS		6.76	5.80
NMS total score		10.39	5.03
MDS-UPDRS III		25.87	14.09
PDQ-39 mobility		44.64	27.67
PDQ-39 activities of daily living		34.71	25.40
PDQ-39 emotional well-being		32.84	21.67
PDQ-39 stigma		21.77	20.42
PDQ-39 social support		17.28	19.28
PDQ-39 cognition		33.14	19.79
PDQ-39 communication		27.88	20.44
PDQ-39 bodily discomfort		38.60	23.62
PDQ-39 summary index		31.36	16.12

BDI, Beck Depression Inventory; HRQoL, health-related quality of life; MDS-UPDRS, Movement Disorder Society-sponsored revision of the Unified Parkinson’s Disease Rating Scale; MoCA, Montreal Cognitive Assessment; NMS-Q, non-motor symptoms questionnaire; PD, Parkinson’s disease; PDQ, Parkinson’s disease questionnaire; SAMS, Stendal Adherence with Medication Score.

## Data Availability

The datasets generated for this study are available from the corresponding author on request.

## Supplementary Materials

The following are available online at https://www.mdpi.com/2076-3425/11/2/273/s1, Table S1: Multivariable linear regressions, Table S2: Comparison of existing cross-sectional studies on adherence and health-related quality of life.
