# Supplementary material for: Self-Reported Nonadherence to Medication Is Not Associated with Health-Related Quality of Life in Parkinson’s Disease
Source: Brain Sci. 2021 Feb 22;11(2):273. doi: 10.3390/brainsci11020273 (PMC7926683; doi:10.3390/brainsci11020273)
Supplement: Supplementary file 1 [file brainsci-11-00273-s001.pdf]

Self-report nonadherence to medication is not associated with health-related quality of life in Parkinson´s disease

Supplementary Table 1

S1: Multivariable linear regressions.

Predictors	Unstandardized coefficients		Standardized coefficients	*t*	*p*
	b	SE	β		
Predictors of the SAMS (corrected R^2 = 0.13)					
Constant	10.56	3.06		3.5	0.001
Female gender	− 2.55	0.87	0.35	− 2.9	0.004
MoCA	− 0.31	0.11	0.34	− 2.8	0.005
NMS-Q	0.15	0.09	0.11	1.6	0.107
Number of medications per day	0.17	0.11	0.11	1.6	0.112
BDI	0.11	0.07	0.09	1.5	0.14
Predictors of PDQ-39 cognition domain (corrected R^2 = 0.48)					
Constant	13.4	7.37		1.8	0.07
NMS-Q	2.28	0.25	0.84	9.1	< 0.001
BDI	0.66	0.19	0.13	3.5	< 0.001
MoCA	− 0.51	0.27	0.04	− 1.9	0.065

BDI, Beck Depression Inventory; MoCA, Montreal Cognitive Assessment; NMS-Q, non-motor symptoms questionnaire; PDQ, Parkinson’s disease questionnaire; SAMS, Stendal Adherence with Medication Score.

Supplementary Table 2

S2: Comparison of existing cross-sectional studies on adherence and health-related quality of life

Study	Grosset et al. 2005	Grosset et al. 2009	Straka et al. 2019	Our study
Design	Single-center Cross-sectional	Multicenter Cross-sectional	Single-center Cross-sectional	Single-center Cross-sectional
N	54	112	124	164
Age (years) (mean, SD)	61.9 (11)	65 (10)	68 (11.8)	71 (9)
MDS-UPDRS III (mean, SD)	27 (13)	20 (14–27)	30.5 (10)	25.8 (14)
Disease duration (years) (mean, SD)	5.4 (3.9)	7.7 (8.2)	7 (5)	9.3 (4.3)
Depression	GDS 10.4 (7)	GDS 7 (4-12)	GDS 8 (8)	BDI = 11.4 (6.6)
Cognition	MMSE 28 (2)	MMSE 29 (28-30)		MoCA 22.5 (4.2)
Adherence measure	Electronic monitoring bottles	Electronic monitoring bottles	8-Item Morisky Medication Adherence Scale	SAMS
HRQoL measure	PDQ-39	PDQ-39	PDQ-8	PDQ-39
PDQ-39 SI (mean, SD)	29 (17)	17 (10–28)	8 (8)	31.4 (16.1)
Association with nonadherence	All 8 PDQ-39 domains (strongest association for social support)	MDS-UPDRS III and PDQ-39 mobility domain	Univariate correlation with PDQ-8; no association in multivariable regression	Univariate correlation with PDQ-39 domain cognition; no association in multivariable regression

BDI, Beck Depression Inventory; GDS, Geriatric Depression Scale; HRQoL, health-related quality of life; MDS-UPDRS, Movement Disorder Society-sponsored revision of the Unified Parkinson's Disease Rating Scale; MMSE, Mini-Mental State Examination; MoCA, Montreal Cognitive Assessment; PDQ-39 SI, Parkinson’s disease questionnaire (39 items) summary index; SAMS, Stendal Adherence with Medication Score.
